# How to Evaluate Hospital Care in the Dying Phase—Development of a Data Extraction Tool for Retrospective Medical Record Analysis

**DOI:** 10.1111/jep.70174

**Published:** 2025-08-13

**Authors:** Sukhvir Kaur, Sophie Meesters, Aneta Schieferdecker, Annika Dangendorf, Barbara Strohbücker, Nikolas Oubaid, Anneke Ullrich, Viola Milke, Karin Oechsle, Holger Schulz, Raymond Voltz, Kerstin Kremeike

**Affiliations:** ^1^ Department of Palliative Medicine, Faculty of Medicine and University Hospital Cologne University of Cologne Cologne Germany; ^2^ Palliative Care Unit, Department of Oncology, Hematology and BMT University Medical Center Hamburg‐Eppendorf Hamburg Germany; ^3^ Department of Nursing, Medical Center of Cologne University of Cologne Cologne Germany; ^4^ Department of Medical Psychology University Medical Center Hamburg‐Eppendorf Hamburg Germany; ^5^ Center for Integrated Oncology Aachen Bonn Cologne Duesseldorf (CIO ABCD), Faculty of Medicine and Cologne University Hospital University of Cologne Cologne Germany; ^6^ Center for Health Services Research (ZVFK), Faculty of Medicine and Cologne University Hospital University of Cologne Cologne Germany

**Keywords:** dying in hospital, dying phase, medical record analysis, palliative care, quality of care

## Abstract

**Background:**

Hospitals are the most common place of death in European countries, including Germany, where nearly half of the population dies in hospitals, mostly outside specialised palliative care wards. At the same time, quality of hospital care in the dying phase is reported as poor. Although existing (inter‐)national guidelines provide outcome variables, their evaluation of implementation is lacking. This study aims to develop and test a structured tool for data extraction from medical records (MRs) to evaluate hospital care in the dying phase. The provision of such a tool can help to identify needs for improvement of care.

**Methods:**

We developed a data extraction tool by operationalizing recommendations for the dying phase of the evidenced‐*based German National Palliative Care Guideline*. The tool was used to extract notes from MRs of *n* = 400 deceased patients of 10 general wards and intensive care units at two University Medical Centres. We analysed the tool's information density and content validity. Descriptive statistics were calculated as frequencies and percentages.

**Results:**

The final tool consists of 39 variables in six domains. Initially, 55 variables were derived from guideline recommendations. With regard to content validity, notes for 37 (67%) variables could be extracted from the MRs, while 16 variables were removed due to poor or unclear documentation. Two additional variables were identified inductively and included in the final tool. Notes could be extracted for all domains, while information density (% of MR with notes) varied: (1) Dying process and death (*n* = 380, 95.0%), (2) Medication and interventions (*N* = 323, 80.7%), (3) Information and involvement of patients and informal caregivers (*n* = 155, 38.8%), (4) Symptom assessment (*n* = 105, 26.3%), (5) Involvement of specialised palliative care (*n* = 78, 19.5%), (6) Goals‐of‐care (*n* = 76, 19.0%). Variation in documentation can reflect differences in care provision or recording practices, suggesting a need for documentation standards.

**Conclusion:**

The tool enables a structured retrospective analysis of guideline‐recommended aspects of care in the dying phase in MRs, applicable to both general wards and intensive care units. It can support quality improvement by identifying documentation gaps and areas of care improvement, and can contribute to target interventions in different hospital settings. To obtain a comprehensive understanding of the care provided, MR analysis should be combined with other methods and perspectives and tested in other settings.

**Trial Registration:**

The study is registered in the German Clinical Trials Register (DRKS00025405).

**Abbreviations**
DNI: do‐not‐intubate
DNR: do‐not‐resuscitate
EC: expert consensus
IC: informal caregiver
ICU: intensive care unit
MR: medical record
MRA: medical record analysis
QI: quality indicators

## 1 Background

A significant proportion of the population in European countries dies in hospitals making them an important setting for end‐of‐life care [1]. In Germany, 47% of all deaths occur in hospitals, mostly outside specialised palliative care wards [2, 3, 4]. At the same time, the hospital is the least preferred place of death for both, patients and their informal caregivers (IC) [5]. Care in the dying phase in hospitals outside of palliative care wards is reported to be poor [6, 7, 8]. For example, the onset of the dying phase outside of palliative wards is often recognised too late [9]. Communication about the dying process is reported to be poor, life‐sustaining treatment is also often inappropriate, and pain and symptom management insufficient [6]. Patients continue to receive life‐sustaining treatments such as mechanical ventilation or dialysis despite an indicated change in goals‐of‐care or signs of the beginning of the dying phase [10]. IC may not be informed in time to be present at the patient's death or to say goodbye [11]. These findings contribute to the perception of poor quality care in the dying phase and highlight the need for structured evaluation and improvement.

Several guidelines have been published that define recommendations for high quality of care in the dying phase [9, 12, 13, 14, 15]. At the international level, the *AMBER Care Bundle* focuses on care for patients with uncertain recovery and aims to improve decision‐making, joint communication, and care planning [15]. The *Best Care for the Dying Patient* recommendations focus on symptom control, communication, psychosocial and spiritual support in the last days of life [12]. In Germany, the national Guideline *Palliative Care for patients with incurable cance*r has been developed and is applicable beyond the oncological setting [9]. It includes evidence‐ and consensus‐based recommendations and quality indicators (QI), among others also on care in the dying phase [9].

An assessment of the current care provision can serve as a first step in optimising care in the dying phase [16]. It enables the identification of areas with need for improvement and subsequently supports the implementation and evaluation of interventions [17]. There are well‐known challenges in directly assessing dying patients' experiences directly as they are burdened and In a reduces functional status [18, 19, 20]. Therefore, most studies use retrospective proxy report by health care professionals or ICs [17, 20]. This can be done using qualitative and quantitative methods. A widely used quantitative method that allows generalisation and comparison of results across large numbers of patients is medical record analysis (MRA) [21, 22]. Retrospective MRA is often used where a prospective approach is not feasible due to ethical considerations mentioned above [23, 24]. Medical records (MRs) can offer information on health care delivery and clinical information [25, 26]. Studies measuring the quality of end‐of‐life care in hospitals outside specialist palliative care ward by MRA already exist [18, 27, 28, 29, 30, 31, 32, 33, 34, 35, 36, 37, 38, 39]. They assessed clinical information like recognition of the dying phase, (life‐sustaining) treatment decisions, the existence of Do‐Not‐Resuscitate/Do‐Not‐Intubate (DNR/DNI) orders and symptom management. Only one study of the above cited evaluated the documentation of conversations with IC [30]. The cited studies focused on patients suffering from specific diseases or belonging to certain age groups. Also, most existing studies used self‐developed data extraction tools without clearly describing their development process or systematically linking them to evidence‐based frameworks. This lack of standardised, guideline‐based tools limits the assessment of care quality and the generalisability and comparability of results. To our knowledge, no validated tool exists that enables structured retrospective analysis of care in the dying phase across hospital settings based on national guideline recommendations, regardless of hospital department type or patient group. Therefore, the aim of the presented study is 1) the development and 2) application of an evidence‐based data extraction tool for MRA of patients who deceased on general wards and intensive care units (ICU). For this purpose, we operationalized recommendations and QI of the German *Palliative Care* Guideline [9].

## 2 Methods

**Figure 1:**
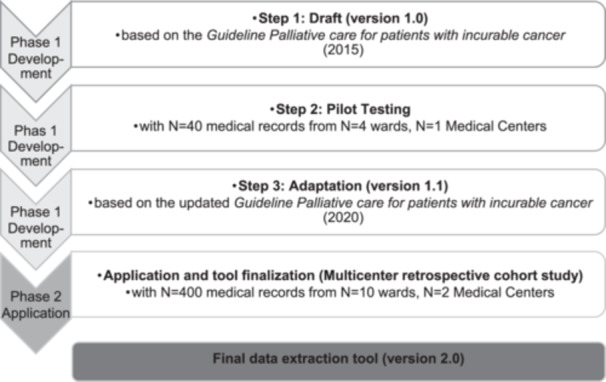
Development and application of a data extraction tool for the retrospective medical record analysis of deceased patients.

As part of a multicenter retrospective cohort study [40], a data extraction tool was developed (phase 1) in three steps (version 1.1). The tool was then applied to extract data from MRs of *n* = 400 patients' deceased on *n* = 10 wards of two medical centres (phase 2). We analysed the information density and content validity of the extracted MR notes (see Figure 1). Based on the results, we finalised the data extraction tool (version 2.0).

### 2.1 Phase 1 Development of Data Extraction Tool

#### 2.1.1 Step 1: Draft (Version 1.0)

The data extraction tool (version 1.0) was drafted within a pilot project that aimed to assess the care for dying patients at one medical centre [41]. The 2015 *German Palliative Care Guideline* is structured in eight chapters and contains key recommendations and 10 QIs [41, 42]. Two authors (AD, BS) operationalized 32 variables based on 3/11 QIs and recommendations regarding care in the dying phase of four guideline chapters: *Dying phase* (16/45), *Breathlessness* (1/21), *Cancer Pain* (3/43) and *Communication* (5/22). Variables were operationalized if they pertained to hospital care in the dying phase and the required information was potentially available in a patient's MR. The draft was organised into six domains (version 1.0) (see Table 1).

**Table 1:** Overview of operationalization sources (recommendations and quality indicators) and tool structure (domains, sub‐domains and variables) from data extraction tool versions 1.0 and 1.1.

		Drafted tool (version 1.0) (2016)	Adapted tool (version 1.1) (2021)
		Based on the 2015 Guideline version, piloted in one medical centre	Based on the 2020 Guideline version, adapted for two medical centres
German Palliative Care Guideline (2015/2021)	Recommendations	25/131	38/136
	Quality indicators	3/10	4/11
Tool structure	Domains	6	7
	Sub‐domains	/	20
	Variables	32	55

As an example of how guideline recommendations were translated into measurable variables, the recommendation stating that ‘tumour‐specific medications and interventions should be discontinued in the dying phase’ was operationalized by identifying any documentation of such treatments—for example, chemotherapy, immunotherapy, or targeted therapy—within the last 72 h of life. Rather than a binary coding, we applied a four‐category coding system to reflect the clinical course (1) not planned (2) stopped, (3) started, and (4) continued. This allowed for a more differentiated analysis of practice patterns in relation to guideline recommendations.

#### 2.1.2 Step 2 Pilot

The draft was piloted with *N* = 40 MRs of patients deceased on four different non‐ palliative care wards (internal ICU, nephrology, urology, visceral surgery). The last 10 patients deceased before 1 April 2016 were selected on each ward. Inclusion criteria were age ≥ 18 and death as reason for discharge from the ward. MRs were available electronically from one and printed from three wards. As the guideline defines the dying phase as the last three to 7 days of patients' life [9], two researchers (AD, BS) extracted notes from MRs related to patients' last three days of life into an Microsoft excel sheet. Some variables extend to the last 7 and 14 days, when recording information about chemotherapy or radiation. The draft appeared to provide a useful basis for a further development of the tool for application in a broader context.

#### 2.1.3 Step 3 Adaptation (Version 1.1)

For use in a multicenter retrospective cohort study for optimising hospital care in the dying phase [40], we used this first draft. In light of the 2020 updated version of the German Palliative Care Guideline, the draft was adapted and extended [9]. The updated version is structured in 16 chapters. Updated and new recommendations were operationalized and existing operationalizations from steps 1 and 2 were adapted through regular consensus discussions among five researchers (SK, KK, NO) including one physician with palliative care expertise and clinical experience (AS) and one psycho‐oncologist with clinical experience (AU) from two medical centres. Overall 55 variables were operationalized based on 4/11 QIs and recommendations regarding care in the dying phase of five guideline chapters: *Dying phase* (30/46), *Breathlessness* (1/18), *Cancer Pain* (3/43), *Communication* (5/19), and *Principles of Palliative Care* (1/10).

The adapted tool (version 1.1), presented as a Microsoft excel sheet, contains patients' demographic and clinical characteristics (*n* = 11 variables) and *n* = 55 variables on hospital care in the dying phase. The variables were structured into *n* = 20 sub‐domains and *n* = 7 domains for improved manageability (see Table 1 and Appendix SA). Variables mostly refer to the last 3 days of patients' lives, with some extending to the last 7 and 14 days (see Appendix SA).

The adapted tool (version 1.1) was pre‐tested using *n* = 3 MRs of the study sample. Therefore, seven researchers from both medical centres extracted notes from the same MRs in three consecutive rounds to test interrater‐reliability. After discussing discrepancies, subsequent adjustments finally yielded consensus across all users of the tool (version 1.1).

### 2.2 Phase 2 Application and Tool Finalisation (Multicenter Retrospective Cohort Study)

#### 2.2.1 Sample

The adapted tool (version 1.1) was applied to the MRs of *N* = 400 deceased patients from *N* = 10 wards (*n* = 40 MRs each) in two medical centres. We aimed to include wards without specialisation in palliative care that regularly care for dying patients, ensuring broad heterogeneity. Participating wards included six ICUs (cardiological, cardiothoracic surgical, internal, 2x surgical) and four general wards (gastroenterology, neurology, nephro‐, endocrino‐ & rheumatology, radiotherapy). These wards differed in annual death numbers (range of means 2019–2022: 11‐201) and bed capacity (12–30). *N* = 6 wards used electronical and *N* = 4 wards paper‐based MRs. Patients who died between January 2018 and August 2021 were included by randomised sampling. Depending on the numbers of death, we either drew a random sample or carried out a full collection of *n* = 40 patients for each ward, with *n* = 20 patients who died before and after the outbreak of the COVID‐19 pandemic respectively (reference date: 11.03.2020). Inclusion criteria were age ≥ 18 and death as the reason for end of care in this ward.

#### 2.2.2 Data Collection

On each ward, staff members of the participating hospital wards provided the printed and anonymized MRs to the research team. Six researchers transferred notes regarding the variables from the MRs into the data extraction tool from September 2021 until December 2022. During this time, four different procedures were applied to achieve a common understanding of the variables and to reduce extraction errors:

1. Researchers extracted data from a randomly selected 20% of MRs [43, 44], either jointly or individually and comparing results afterwards.
2. As recommended [42], a detailed manual for data extraction and a medication list were developed and continuously updated and used.
3. Regular cross‐site meetings were scheduled to address questions and uncertainties during the extraction process.
4. Joint MR extraction and consultation with specialist palliative care expertise occurred as needed, for example, to answer questions on medication [43].

#### 2.2.3 Data Analysis

To assess the applicability of the adapted tool (version 1.1), we analysed the generated data on domain and sub‐domain level. We examined information density quantitatively and content validity qualitatively [44]. Both, information density and content validity, indicated which guideline‐based variables could realistically be assessed using MR documentation. For information density, we calculated the number and percentage of MRs where we found notes for all variables, at least for one variable (party) or no variable in the sub‐domain. Content validity was assessed through a structured, iterative consensus process. First, the operationalized guideline recommendations were independently reviewed by a multidisciplinary team consisting of three scientists experienced in palliative care research (KK, SM, SK) and one palliative care physician (AS). Each team member assessed whether the operationalized variables clearly and accurately reflected the specific guideline recommendations. The team then met repeatedly to discuss discrepancies in their assessments of variables' alignment with guideline recommendations until consensus was reached on the inclusion, adjustment or removal of variables. Variables were retained only if the team could agree on a clear, unambiguous link to the guideline recommendations. Data processing and analysis was performed using RStudio 4.12.

## 3 Results

We developed and applied a data extraction tool in two phases: (1) by operationalizing recommendations regarding care in the dying phase of the German *National Palliative Care Guideline* into variables and (2) extracting data from MRs of *n* = 400 deceased patients of 10 non‐palliative wards at two medical centres. Data from MRs could be extracted for all seven domains of the tool (version 1.1). However, information density (see Table 2) and content validity (see Table 3) of generated data varied considerably within the domains.

**Table 2:** Information density on (sub‐)domain‐level for version 1.1 (*N* = 400 MRs, *N* = 10 wards, *N* = 2 medical centres).

Information density	Number of variables	Notes found in MRs		
		Yes^a^	Partly^b^	No
		*N* (%)	*N* (%)	*N* (%)
Domain 1: Dying process and death		380 (95.0)		20 (5.0)
*1.1 Nursing notes*	4	20 (5.0)	356 (89.0)	24 (6.0)
*2.2 Physicians’ notes*	4	48 (12.0)	336 (84.0)	16 (4.0)
Domain 2: Medication and interventions		323 (80.7)		
*2.1 Monitoring and life‐sustaining Interventions*	8	/	397 (99.2)	3 (0.8)^c^
*2.2 Medication for symptom control*	2	357 (89.2)	9 (2.3)	34 (8.5)
*2.3 Sedation*	2	2 (0.5)	123 (30.7)	275 (68.8)
*2.4 Artificial nutrition & hydration*	3	62 (15.5)	304 (76.0)	34 (8.5)^c^
Domain 3: Information and involvement of patients and informal caregivers		155 (38.8)		
*3.1 Patients' information about impending death*	3	5 (1.3)	46 (11.5)	349 (7.2)
*3.2 Informal caregivers’ information about patients' impending death*	3	56 (14)	241 (60.2)	103 (5.8)
*3.3 Involvement and support of informal caregivers*	2	15 (3.8)	73 (18.2)	312 (8.0)
*3.4 Involvement and consideration of patients' wishes*	3	1 (0.2)	99 (24.8)	300 (5.0)
*3.5 Shared decision making*	3	47 (11.7)	238 (9.5)	115 (8.8)
*3.6 Informal caregivers' presence at patients' death*	2	10 (2.5)	180 (5.0)	210 (2.5)
*3.7 Information of informal caregivers about patients' death (deleted)*	2	89 (22.2)	48 (12.0)	263 (5.8)
*3.8 Steps after patients' decease (version 1.1)* *Indications after death (version 2.0)*	2	26 (6.5)	68 (17)	306 (76.5)
Domain 4: Symptom assessment		105 (26.3)		
*Assessment of pain and other symptoms*	3	51 (12.7)	94 (23.5)	255 (63.8)
Domain 5: Involvement of specialised palliative care		78 (19.5)		
*Hospital palliative care support team*	1	78 (19.5)	0	322 (80.5)
Domain 6: Goals‐of‐care		706 (19.0)		
*6.1 Change of goals‐of‐care*	7	27 (6.8)	67 (16.7)	306 (6.5)
*6.2 Holistic approach (deleted)*	1	58 (14.5)	0	342 (5.5)
Domain 7: Continuity of care (deleted)				
*7.1 Number of nurses*	1	373 (3.2)	0	27 (6.8)
*7.2 Number of physicians*	1	376 (94.0)	0	24 (6.0)

*Note:* Percentage and number of MRs for which notes were found for the variables on (sub‐)domain level. Domains are arranged in descending order to their information density and the average percentage of MRs with notes for at least one variable is displayed. On sub‐domain level the table shows the number and percentage of MRs for which notes for all variables was found (yes), for at least one variable was (partly) was found and for which no notes were found (no).

^a^ Information was found for all variables in the sub‐domain.

^b^ Information found for at least one variable in the sub‐domain.

^c^ No notes found that interventions were conducted.

**Table 3:** Changes to the adapted tool after the application phase (number of variables and sub‐domains).

			Types and reasons for change					
	Number of variables		Variable deleted					Variable added
(Sub‐)Domain	Version 1.1	Version 2.0	Lack of documentation	Inadequate operationalization	Clinically not relevant	Notes moved to another domain	Variable aggregated within	Operationalization possible
**Domain 1: Dying process and death**								
*1.1 Nursing notes*	4	1			1	2		
*1.2 Physicians' notes*	4	1			1	2		
**Domain 2: Medication and interventions**								
*2.1 Monitoring and life‐sustaining interventions*	8	8	
*2.2 Medication for symptom control*	2	2	
*2.3 Sedation*	2	2	
*2.4 Artificial nutrition and hydration*	3	2		1	
**Domain 3: Information and involvement of patients and informal caregivers**								
*3.1 Patients' information about impending death*	3	1	2	
*3.2 Informal caregivers' information about patients' impending death*	3	2	1	
*3.3 Involvement and support of informal caregivers*	2	2	
*3.4 Involvement and consideration of patients' wishes*	3	1		1		1		
*3.5 Shared decision making*	2	3		1
*3.6 Informal caregivers' presence at patients' death*	3	1	
*3.7 Information of informal caregivers about patients' death (deleted)*	2	0		1		1		
*3.8 Steps after patients' decease (version 1.1)* *Indications after death (version 2.0)*	2	1		1	
**Domain 4: Symptom assessment**								
*Assessment of pain and other symptoms*	3	3	
**Domain 5: Involvement of specialised palliative care**								
*Hospital palliative care support team*	1	1	
**Domain 6: Goals‐of‐care**								
*6.1 Change of goals‐of‐care*	6	7		1
*6.2 Holistic approach (deleted)*	1	0		1	
**Domain 7: Continuity of care (deleted)**								
*7.1 Number of nurses*	1	0		1	
*7.2 Number of physicians*	1	0		1	
**Total Number**	**55**	**39**	**−3**	**−6**	**−2**	**−6**	**−1**	**+2**

The content of the extracted notes showed that 37 out of 55 variables (67%) represented the guideline recommendation. Due to low or unclear documentation, 16 of 55 initially operationalized variables (32%) were removed. We added two variables inductively during the extraction process, as notes provided more information than expected. Concrete examples of variables and reasons for their removal are listed below at domain level. For a summary of all changes to the adapted tool please refer to Table 3. The final data extraction tool (version 2.0) consists of *n* = 6 domains, *n* = 18 sub‐domains and *n* = 39 variables (see Table 3 for the changes, the (number of) variables and sub‐domains. Appendix SA shows the final tool).

### 3.1 Domain 1: Dying Process and Death

For this domain, highest information density was analysed (95% of *n* = 400 MRs). The domain consists of two sub‐domains (*1.1 Nursing notes* and *1.2 Physicians' notes*). We operationalized four variables respectively for each sub‐domain and made following changes:

- Treatment decisions and interventions in the dying phase should be documented and continually re‐assessed (Expert consensus (EC) 19.6), we collected this information within the variable ‘re‐evaluation of treatment measures’. Notes showed that especially in ICUs, re‐evaluation is often not possible as death occurs very soon after change of interventions or goals‐of‐care. Therefore, notes were moved to the **domain 6 Goals‐of‐care** and the variable was deleted from both sub‐domains.
- According to the guideline recommendation containing criteria to assess whether the dying phase has begun, for example, changes in breathing, skin, emotions and consciousness (EC 19.1), we collected this information within the variable ‘dying process’. Notes in both sub‐domains reflect content of guideline recommendation.
- Regarding the variable ‘moment of patient's death’, notes contained no further information besides the time of patient's death. As this date is already recorded in the demographic data, the variable is deleted from both sub‐domains.
- Regarding notes in the variable ‘information after patient's death’, notes mostly contained ICs' notification of patients' death. Death notifications were also extracted in the variables in sub‐domain *3.8 Steps after patients' decease (Re‐named Indications after death)*. Notes were moved to *3.8* and the variable was deleted.

### 3.2 Domain 2: Medication and Interventions

Notes on this domain were extracted from 80.7% of MRs. The domain consists of four sub‐domains and 15 variables. After assessing notes on this domain, following changes were made on variable‐level:

- *Monitoring and life‐sustaining interventions (99.2%):* according to guideline recommendation stating that life sustaining interventions should be stopped in the dying phase (EC 19.31–19.36), we extracted notes on continuation/stopping of vital signs and blood glucose monitoring, oxygen therapy, mechanical ventilation, dialysis/hemofiltration, tumour‐specific therapy, artificial nutrition and hydration and antibiotic therapy and deactivation of an implanted cardioverter‐defibrillator within eight variables. All variables reflect content of guideline recommendation.
- *Medication for symptom control (91.5%):* drug substances from the groups of opioids, antipsychotics, benzodiazepines and anticholinergics should be started or continued (EC 19.31), therefore we extracted this within two variables. Both of them reflected guideline recommendation.
- *Sedation (31.2%):* according to guideline recommendation, palliative sedation should be carried out by competent physicians and nurses experienced in palliative care (EC 19.37). We extracted this information in two variables, assessing whether palliative sedation or deep continuous sedation was carried out.
- *Artificial nutrition and hydration (86.5%):* according to guideline recommendation EC 19.38, patients' need for artificial nutrition and hydration should be assessed on an individual basis. We extracted this information within the variable ‘needs assessment for nutrition and hydration'. The content of notes showed that this information was not sufficiently documented. Therefore, this variable was deleted, two variables remain.

### 3.3 Domain 3: Information and Involvement of Patients and Informal Caregivers

Notes were extracted from 38.8% of MRs. The domain contains of eight sub‐domains. Following changes on variable‐level were made per sub‐domain:

- *Patients' information about impending death (12.8%) and 3.2. ICs’ information about patients' impending death (74.2%):* as the dying patient and their IC should be adequately informed about the impending death and expected changes in the dying phase (EC 19.7), we operationalized three variables on patients and ICs being informed (1) about the impending death, (2) about expected changes in the dying phase, and (3) their reactions on the impending death. Notes on (2) und (3) were lacking of documentation, therefore they were deleted from the tool; one variable remains, respectively.
- *Involvement and support of informal caregivers (22%):* as ICs should receive offers of support (EC19.8), we operationalized two variables assessing their wishes/resources and support offers. Both of them reflected guideline recommendation.
- *Involvement and consideration of patients' wishes (25%):* as treatment decisions and measures in the dying phase shall be in accordance with the needs of the dying patients and ICs (EC 19.5), we operationalized this recommendation in three variables: (1) patients' verbalised wishes, (2) patients' non‐verbalised wishes, feelings and needs through various indicators like facial expressions, gestures, and body language (EC 19.9), (3) involvement of wishes in further treatment. Notes in (2) lacked selectivity compared to notes in **Symptom assessment (domain 4)** and were moved there. Extracted notes in (3) did not adequately reflect clinical practice due to inadequate operationalizability of the guideline recommendation. Consequently, both variables were deleted from the tool.
- *Shared decision‐making (71.2%):* useful notes on patients' documented wishes, in documents as living will or communicated by ICs, were found in the MRs and operationalized in an additional variable as it reflects guideline recommendations (EC 4.7, 19.5).
- *Presence of informal caregivers at patients' death (47.5%):* Two variables assessed whether (1) ICs were present when the patient died and if they were not present, were able to say goodbye (EC 19.10). Both variables reflect guideline recommendation.
- *Information of ICs about patient's death (32.4%):* ICs should be informed of patients' death in a sensitive and timely manner (EC 19.39). Two variables assessed (1) if there were any notes about the person that informed the ICs about the patient's death and (2) the circumstances of this information. The notes on both variables showed that only physicians informed ICs about patients' death in person. As these variables did therefore not add any further information, the sub‐domain was deleted from the tool.
- *Steps after patient's death (23.5%):* ICs shall be allowed to say goodbye in accordance with their needs and resources, cultural practices and religious duties (EC 19.40). Therefore, we created two variables to assess (1) whether there are any notes that ICs were informed about the next steps after patient's death and (2) if they were able to say goodbye in accordance to their needs and resources. Notes from both variables showed that they were not selectively from each other. Therefore, variables were merged into one and the sub‐domain was renamed into *indications after death*. As described above, notes from Domain 1 (variable ‘information after patient's death’) were added to the sub‐domain.

### 3.4 Domain 4: Symptom Assessment

Notes were extracted from 26.3% of MRs. The guideline states that assessment of pain intensity and other symptoms should be carried out by the health care team for example, by use of one‐dimensional pain scales (EC 9.1–9.3). We operationalized three variables assessing if a symptom assessment was carried out, which symptoms were assessed and if an assessment tool was used. Content of extracted notes reflects guideline recommendations. As mentioned above, notes from the sub‐domain *3.4 Involvement and consideration of patients' wishes in the dying phase* were moved to this domain.

### 3.5 Domain 5: Specialist Palliative Care

As patients from non‐palliative care wards can receive specialist palliative care for example, through a hospital palliative care support team, we operationalized a variable assessing how many times the hospital palliative care support team was involved in patient treatment (QI 11). Notes were extracted from 19.5% of MRs, indicating low information density in comparison to other domains. However, notes reflected the content of guideline recommendation; therefore, no changes were made.

### 3.6 Domain 6: Goals‐of‐Care

Lowest information density was analysed for domain 6 (19% of MRs). The domain contains of two sub‐domains, following changes were made:

- *Change of goals‐of‐care (24.5%):* notes on time of changes of goals‐of‐care and information weather escalation of therapy, life‐sustaining therapy or symptom‐oriented therapy has been determined (EC 6.15–6.19) were extracted for six variables. The time of DNR/DNI orders were also found in MRs and operationalized in an additional variable as it reflects guideline recommendations (EC 4.7).
- *Holistic approach (14.5%):* the recommendations states that all dimensions of quality of life should be considered (EC 19.4, 19.30). Therefore, the operationalized variable extracted notes that other dimensions (social, psychological, spiritual) beyond the physical one were considered in care decisions and interventions. Notes did not adequately reflect the guideline recommendation. Consequently, the sub‐domain and its variable were deleted from the tool.

### 3.7 Domain 7: Continuity of Care (Deleted)

According to the guideline, dying patients should be supported with continuity of care (EC 19.16, 19.27). Therefore, we extracted the number of physicians and nursing staff that were involved in the patients' care were extracted from 93.6% of MRs. However, the notes lacked information regarding the continuity of staff, making it unclear whether the same HCP consistently participated in care. The domain was deleted from the tool as notes did not adequately reflect clinical practice due to inadequate operationalizability of the guideline recommendation.

## 4 Discussion

We developed a data extraction tool to assess hospital care in the dying phase from MRs of deceased patients using evidence‐based variables derived from the *German National Palliative Care Guideline* [9]. The tool consists of a comprehensive set of 39 variables structured into *n* = 18 sub‐domains and *n* = 6 domains, completed by *n* = 11 variables regarding clinical and demographic characteristics. The tool proved to be feasible to extract notes for all relevant domains of care in the dying phase. However, information density and content validity varied considerably within the domains.

### 4.1 Reflection of Guideline Recommendations in Medical Records

The tool enables to analyse if QIs and recommendations on care in the dying phase are represented in MR documentation. However, analysis of information density showed that MRs did not consistently reflect all relevant guideline recommendations. We were able to extract more notes for objective and quantifiable variables (e.g., medication and interventions) rather than subjective variables (e.g., related to information, care of and communication with patients and their ICs). Domains such as ‘symptom assessment’ and ‘goals‐of‐care’ had significantly lower documentation density than domains such as ‘dying process and death’. One possible reason for this is that the documentation of the process and stage of dying includes formal and routine documentation requirements, such as recording the time of death. In contrast, symptom assessments and changes in goals‐of‐care are less formally embedded in documentation processes—particularly outside of palliative care settings. Moreover, these aspects require communication and reflection processes that may not be consistently undertaken or documented, particularly in acute care settings with high workload and limited training in end‐of‐life care [6]. Our data thereby supports previous study results showing that objective data, such as vital sign monitoring, is more reliably documented than subjective free‐text data [45], suggesting potential gaps in medical record documentation and care delivery.

One of the foremost challenges in documentation of the patients last days of life lies in the different perception of high‐quality end‐of‐life‐care among health care professionals, especially in the area of communication and information [45]. Studies showed that care documentation in acute settings are incomplete and lacking in continuity with little emphasis on psychosocial aspects of care. This variation impacts the way information is documented by different healthcare professionals in MRs, potentially influencing the reliability of data obtained from MRs [26]. Our findings raise the question whether healthcare professionals prioritise documenting aspects such as medication and interventions over conversations with patients and their IC. The lack of a standardised documentation standard limits the assessment of care by MRs [42]. Implementation of documentation standards could reduce documentation of avoidable interventions in MRs and focus on the aspects emphasised in the guideline recommendations [45].

Furthermore, the operationalization of some variables proved challenging, for example, holistic approach in patient care, highlighting the difficulty in operationalization of qualitative outcomes that allow for more interpretation [46]. This emphasises the need for more operationalization of subjective aspects during MRA [46]. A manual and regular meeting between data extractors enables subjective aspects to be assessed more reliably. Therefore, variables have been selected for the tool that can be adequately mapped to match the documentation as well as possible. The decision to operationalise certain recommendations in variables rather than others stem from the need to prioritise aspects of care deemed more measurable within MRs.

### 4.2 Use of Medical Records to Assess Hospital Care in the Dying Phase

The data within MRs were not primarily documented for research purposes and, as a result, may lack both quality and quantity for scientific evaluation/data analysis [47]. It is crucial for data sources intended for research purposes to be both reproducible and valid. This highlights a weakness in the reliability of MRA as a data source [47]. The quality of the MRs can be influenced by a number of factors, for example, paper‐based versus electronic record [48]. On the other hand, unlike methods such as participant observation, where the data collection may influence outcomes, medical record data remains unaffected by the data collection process [46, 49, 50]. MRA is limited to the information documented in MRs and may not offer a comprehensive view of the care delivered to patients [46]. It is important to note that MRA assesses the documentation of care in the dying phase rather than the actual provision of care. Relying solely on record analysis may not present a complete picture, underscoring the importance of additional assessment instruments [51]. Nevertheless, the utilisation of MRs in research can be valuable as they reflect real‐world healthcare practices [46]. MRs have a significant impact on the quality of care provided. Accurate and detailed documentation ensures that healthcare providers have access to essential information, which can lead to more effective decision‐making and improved patient outcomes [45]. Additionally, thorough documentation plays a crucial role in care coordination among different providers, enhancing communication and information flows in the team and continuity of care for patients. As mentioned above, the implementation of documentation standards could foster quality of MR documentation. Comprehensive documentation also serves as a means of accountability, enabling healthcare organisations to demonstrate the quality of care they provide and to identify areas for improvement [39, 52].

The implementation of the tool in clinical practice offers opportunities and challenges. A potential facilitator is the tools’ development based on a national guideline, that may foster acceptance among healthcare professionals and its applicability across different wards. However, potential barriers include limited time and staff resources and variation in documentation cultures across wards and professions. For successful implementation, institutional support, staff training, and integration into existing documentation workflows are essential. Piloting and adapting the tool to the local context could help to further tailor it to the needs of the setting and promote its sustainable use [53].

### 4.3 Strengths and Limitations

To our knowledge, we developed the first standardised data extraction tool built on evidence‐based guideline recommendations and QIs on care in the dying phase instead of self‐selected outcomes [9]. This approach thereby facilitates a holistic overview of hospital care in the dying phase, including not only interventions and medication but also communication and involvement of patients and their ICs. As we used data from ten different non‐palliative wards of two medical centres to develop the tool, we enhance the generalisability of results. The tool is applicable for both general wards and intensive care units, irrespective of the hospital department or the primary disease of the deceased patient, ensuring data comparability across wards.

Limitations relate primarily to the method of MR documentation, as it only provides indications on care provision, as it is not always known what might have happened beyond the documentation. The information gathered from patient record analysis serves to identify areas of care that either meet established standards or require improvement, including details of medication, interventions, and communication. Potential for bias in the interpretation of the free‐text data as well as incomplete or inaccurate documentation exists, influencing validity and reliability of results.

## 5 Conclusion

The results of our study highlight specific documentation gaps in care in the dying phase. To address these gaps, the use of the developed tool may provide useful insights on how to support quality improvement efforts, including structured documentation templates and targeted staff training. The developed tool allows for a structured retrospective analysis of the routine documentation in MRs and the extent to which recommendations and QIs regarding care in the dying phase are reflected. To gain a full understanding of the care provided to patients in this phase, future research could complement MRA with qualitative methods, such as interviews with IC and healthcare professionals [20]. Such a *mixed‐methods* approach could contribute to knowledge about barriers to high‐quality care not captured in MRs and provide insights into how to improve both documentation and quality of care in the dying phase.

## Author Contributions

R.V. is Principal Investigator and responsible for the study design and dissemination. K.K. is responsible for the study design, project management, recruitment, data collection, data analysis and dissemination. K.O., A.U. and H.O. are responsible for the study design, recruitment of wards, data analysis and dissemination. S.K., S.M., N.O., A.U., A.D., B.S., and A.S. are responsible for development of the data extraction tool. S.K., S.M. and N.O. are responsible for data collection, data analysis and dissemination. All authors read and approved the final manuscript.

## Ethics Statement

Ethical approval was obtained from the ethics committee of the Medical Faculty of the University of Cologne on 19.04.2021 (20‐1727) and by the ethics committee of the General Medical Chamber, Hamburg on 03.08.2021 (2021‐200061‐BO‐bet). We collected pseudonymized patient data; therefore, no written or verbal informed consent was needed.

## Conflicts of Interest

The authors declare no conflicts of interest.

## Supporting information

Data Extraction Tool.

## Data Availability

Data available on request due to privacy/ethical restrictions.
