# Supplementary material for: How to Evaluate Hospital Care in the Dying Phase—Development of a Data Extraction Tool for Retrospective Medical Record Analysis
Source: J Eval Clin Pract. 2025 Aug 13;31(5):e70174. doi: 10.1111/jep.70174 (PMC12344475; doi:10.1111/jep.70174)
Supplement: Supplementary file 1 — Data Extraction Tool. [file JEP-31-0-s001.docx]

**Data extraction tool**

| **Demographic data & clinical characteristics** | |
| --- | --- |
| **Items (n=11)** | **Option** |
| 1. How old was the patient, when the patient died? | 18- 115 years |
| 1. What was the patients’ gender? | - Male - Female - Divers |
| 1. Which date of death is documented? | dd.mm.yy |
| 1. When was death recorded? | - 0-4 am - 4-8 am - 8-12 am - 12-4 pm - 4-8 pm - 8- 0 pm |
| 1. On which date was the patient admitted to the ward where the patient died? | dd.mm.yy |
| 1. Which ICD-10 diagnosis group* is the patient most likely to be classified in? | - Maligant neoplasm - Diseases of the circulatory system - Diseases of the nervous system - Diseases of the respiratory system - Diseases of the digestive system - Diseases of the genitourinary system - Endocrine, nutritional or metabolic diseases - Injury, poisoning or certain other consequences of external causes - Certain infectious or parasitic diseases - Mental or behavioural disorders - Covid-19 |
| 1. What was the cause of death? (according to death certificate) | - Infection, sepsis - Heart failure, cardiac arrhythmia - Respiratory failure - Bleeding, haemorrhagic shock - Cerebral cause of death - Other |
| 1. Did the patient die during surgery or after unsuccessful resuscitation? | - Yes, death during surgery - Yes, death after resuscitation - No |
| 1. Was the patient adequately able to communicate? | - Yes - No - Partly |
| 1. Are there indicators of people who are involved in the process besides the patient? | - Informal caregivers - Legal guardian - No |
| 1. Are there any notes that a living will and/or a power of attorney was available for the health care staff? | - Yes - No - Patient able to communicate |

| **Domain 1: Dying process and death** | | |
| --- | --- | --- |
| **Items (n=8)** | **Option** | **Guideline Recommendation Number (EC) or**  **Quality indicator (QI)** |
| - 1. **Nursing notes** | | |
| 1. Are there any notes about the re-evaluation of care decisions and interventions in the dying phase in the nursing notes? | - Yes, once - Yes, several times - No | 19.6 |
| 1. Are there any notes about the dying process in the nursing notes? | - Yes - No - Stopping of intensive care therapy | 19.1  19.2 |
| 1. Are there any notes about the occurrence of death in the nursing notes? | - Yes - No |  |
| 1. Are there any notes about the time after death in the nursing notes? | - Yes - No |  |
| - 1. **Physicians’ notes** | | |
| 1. Are there any notes about the re-assessment of care decisions and interventions in the dying phase in the physician’s notes? | - Yes, once - Yes, several times - No | 19.6 |
| 1. Are there any notes about the dying process in the physician’s notes? | - Yes - No - Stopping of intensive care therapy | 19.1  19.2 |
| 1. Are there any notes about the moment of death in the physician’s notes n? | Yes  No |  |
| 1. Are there any notes about the time after death in the physician’s notes? | - Yes - No |  |

| **Domain 2: Medication and interventions** | | |  |
| --- | --- | --- | --- |
| **Items (n=15)** | **Option** | **Guideline Recommendation Number (EC) or**  **Quality indicator (QI)** | |
| - 1. **Monitoring and treatment** | | |  |
| 1. Was an implanted cardioverter-defibrillator (ICD) deactivated? | - No - Yes - No ICD - No information about de-activation | 19.36 |  |
| 1. Were vital signs routinely measured and documented? | - Not planned - Stopped - Continued - Started - Not reported | 19.33  19.34 |  |
| 1. Was the blood glucose level (routinely) monitored? | - Not planned - Stopped - Continued - Started - Not reported |  |  |
| 1. Was oxygen therapy given? | - Not planned - Stopped - Continued - Started - Not reported | 8.18 |  |
| 1. Was antibiotic therapy given? | - Not planned - Stopped - Continued - Started - Not reported | 19.31  19.33 |  |
| 1. Has dialysis/peritoneal dialysis or hemofiltration been performed?   (7 days prior death) | - Not planned - Stopped - Continued - Started - Not reported | 19.33 |  |
| 1. Was mechanical ventilation been performed? (7 days prior death) | - Not planned - Stopped - Continued - Started - Not reported | 19.33 |  |
| 1. Has chemotherapy/immunotherapy/oral tumour therapy/radiotherapy been administered? (14 days prior death) | - Not planned - Stopped - Continued - Started - Not reported | 19.32  QI 6 |  |
| - 1. **Medication for symptom control** | | |  |
| 1. Were medications of the following substance classes prescribed? | - Opioids - Antipsychotics - Benzodiazepines - Anticholinergics - Multiple answers - No | 19.17  19.28  19.31 |  |
| 1. Were medications of the following substance classes administered? | - Opioids - Antipsychotics - Benzodiazepines - Anticholinergics - Multiple answers - No |  |  |
| - 1. **Sedation** | | |  |
| 1. Was "palliative sedation” carried out? | - Yes - No - Not reported | 19.31  19.37 |  |
| 1. Was deep continuous sedation, that was started before the dying process, carried out? | - Yes - No - Not reported |  |  |
| - 1. **Artificial nutrition and hydration** |  |  |  |
| 1. Are there any notes that the need for artificial nutrition or hydration was assessed? | - Yes - No | 19.38 | |
| 1. Are there any notes that artificial nutrition was administered enteral or parenteral? | - Not planned - Stopped - Continued - Started - Not reported |  |  |
| 1. Are there any notes that artificial hydration was administered? | - Not planned - Stopped - Continued - Started - Not reported |  |  |

| **Domain 3: Information and involvement of patients and informal caregivers** | | |
| --- | --- | --- |
| **Items (n=20)** | **Option** | **Guideline Recommendation Number (EC) or**  **Quality indicator (QI)** |
| **3.1 Patients’ information about impending death** | |  |
| 1. Are there any notes that the patient was informed that the patient is dying? | - Yes - No - Not able to communicate | 19.7 |
| 1. Are there any notes that the patient was informed about changes to be expected in the dying phase? | - Yes - No - Not able to communicate |  |
| 1. Are there any notes how the patient reacted to the impending death? | - Yes - No - Not able to communicate |  |
| - 1. **Informal caregivers’ information about patients’ impending death** | | |
| 1. Are there any notes that the informal caregivers were informed that the patient is dying? | - Yes - No - No informal caregivers | 19.7 |
| 1. Are there any notes that the informal caregivers were informed about changes to be expected in the dying phase? | - Yes - No - No informal caregivers |  |
| 1. Are there any notes on how the informal caregivers reacted on patients impending death? | - Yes - No - No informal caregivers |  |
| - 1. **Involvement and support of informal caregivers** | | |
| 1. Are there any notes that the informal caregivers could participate in the care of the dying patient according to their resources and wishes? | - Yes - No - No informal caregivers | 19.5  19.8 |
| 1. Are there any notes that the informal caregivers received support offers from health care professionals? | - Yes - No - No informal caregivers |  |
| - 1. **Involvement and consideration of patients’ wishes** | | |
| 1. Are there any notes that the patient had the opportunity to talk about wishes or aspects important to him? | - Yes - No - Not able to communicate | 19.5 |
| 1. If the patient has not been able to verbalize:   Are there any notes that the patient's wishes, feelings and needs were observed and assessed through facial expressions, gestures, breathing, muscle tone, eye contact, movement patterns, reactions, and para-verbal sounds? | - Yes - No - Patient able to communicate | 19.9 |
| 1. Are there any notes that the expressed patient’s wishes, principles were considered in the treatment? | - Yes - No |  |
| - 1. **Shared decision making** | | |
| 1. Are there any notes whether the goal-of-care was agreed with the patient? | - Yes - No - Patient not able to communicate | 19.5  4.7 |
| 1. *Are there any notes that the presumed will of the patients who was unable to communicate has been assessed?* | - *Yes, via living will* - *Yes, via informal caregivers* - *Yes, via health care professionals* - *Yes, via legal guardian* - *Yes, multiple answers* - *No* |  |
| 1. Are there any notes whether goals of care were agreed with informal caregivers? | - Yes - No - No informal caregivers |  |
| **3.6 Informal carers’ presence at patients’ death** | | |
| 1. Are there any notes that informal caregivers were present when the patient died? | - Yes - No - No informal caregivers | 19.40 |
| 1. Are there any notes that informal caregivers, that were not present when the patient died, were able to say goodbye? | - Yes - No - No informal caregivers |  |
| - 1. **Information of informal caregivers about patient’s death** | | |
| 1. Are there any notes that informal caregivers were informed the patient’s death? | - Yes, nurse - Yes, physician - Yes, others - No - No informal caregivers | 19.39 |
| 1. Are there any notes about the circumstances of the information about the patient’s death to the informal caregivers? | - Yes - No - No informal caregivers |  |
| - 1. **Indications after death** | | |
| 1. Are there any notes of what happened after patients’ death? | - Yes - No | 19.40  19.41 |
| 1. Are there any notes that the informal caregivers were informed about the next steps after patient’s death? | - Yes - No - No informal caregivers |  |
| 1. Is there information that informal caregivers were able to say goodbye in accordance to their needs and resources after patients’ death? | - Yes - No - No informal caregivers |  |

| **Domain 4: Symptom assessment** | | |
| --- | --- | --- |
| **Items (n=3)** | **Option** | **Guideline Recommendation Number (EC) or**  **Quality indicator (QI)** |
| **Assessment of pain and other symptoms** | | |
| 1. Were symptoms assessed? | - Yes, via clinical evaluation - Yes, via assessment tool - Yes, via both clinical evaluation and assessment tool - No | QI 2  9.1  9.2  9.3  19.12  19.14  19.25  19.26 |
| 1. Please use free text to list the symptoms that were assessed clinically: e.g. anxiety, breathlessness, sleep disturbances, delirium | *Free text* |  |
| 1. Was a symptom assessment carried out? | - Yes, via NRS - Yes, via MIDOS - Yes, via IPOS - Yes, via HOPE - Yes, via VAS - Yes, via VRS - Yes, via Delir Score - Yes, via Smiley-Scale - Yes, via BPS - Yes, via another tool - Multiple answers - No |  |

| **Domain 5: Involvement of specialized palliative care (7 days prior death)** | | |
| --- | --- | --- |
| **Items (n=1)** | **Option** | **Guideline Recommendation Number (EC) or**  **Quality indicator (QI)** |
| Was the hospital palliative care support team involved in the patients’ care? | - Yes - No | QI 11 |

| **Domain 6: Goals-of-care** | | |
| --- | --- | --- |
| **Items (n=8)** | **Option** | **Guideline Recommendation Number (EC) or**  **Quality indicator (QI)** |
| **6.1 Change of goals-of-care** | | |
| 1. Was a change of goal-of-care documented? | - Yes - No - Termination of resuscitation/surgery | 4.7  6.15  6.16  6.17  6.18  6.19  19.43 |
| 1. Are there any notes, that escalation / enouncement of life-sustaining therapy and interventions has been determined? | - Yes - No |  |
| 1. Are there any notes, that de-escalation/enouncement of life-prolonging or -sustaining therapy has been determined? | - Yes - No |  |
| 1. Are there any notes, that escalation or new life-prolonging or -sustaining therapy has been determined? | - Yes - No |  |
| 1. Please use free text to list terms that were used to describe the naming of superordinate concepts, e.g. symptom-orientated therapy, palliative therapy, best-supportive care? | *Free text answer* |  |
| 1. At which time took the change of goal-of-care place? | - 0-23h prior death - 24-47h prior death - 48-72h prior death - >72h prior death - No change of goal-of-care - Not reported |  |
| 1. *At what time was Do-Not-Resuscitate (DNR)/ Do-Not-Intubate established (DNI)?* | - *Before 3 days prior death* - *During the last 3 days prior death* - *No DNR/DNI documented* - *Not reported* |  |
| - 1. **Holistic approach** | | |
| 1. Are there any notes that other dimensions beyond the physical one was considered in care decisions and interventions? | - Yes, following dimensions - No | 19.4  19.30 |

| **Domain 7: Continuity of care** | | |
| --- | --- | --- |
| **Item (n=2)** | **Value** | **Guideline Recommendation Number (EC) or**  **Quality indicator (QI)** |
| - 1. Number of nurses | - 1-x - Not reported | 19.16  19.27 |
| - 1. Number of physicians | - 1-x - Not reported |  |

*Categories according to the International Statistical Classification of Diseases and Related Health Problems, 10th Revision (ICD-10) [ICD-10 Version:2016 (who.int)](https://icd.who.int/browse10/2016/en)

*Added after application phase*

Deleted after
